# Routine transfusion of Rh(D)‐positive RBCs to Rh(D)‐negative patients designated as do not resuscitate conserves Rh(D)‐negative red blood cell inventory

**DOI:** 10.1111/trf.70209

**Published:** 2026-04-12

**Authors:** Julie Katz Karp, Jovanna Everetts, Juliana Guarente, Angelica Vivero, Tiffany Bohr, Mary Harach

**Affiliations:** ^1^ Department of Pathology and Genomic Medicine Thomas Jefferson University Hospital Philadelphia Pennsylvania USA

## Abstract

**Background:**

A minority of blood donors are Rh(D)‐negative, and Rh(D)‐negative red blood cell (RBC) products are often overutilized. As such, Rh(D)‐negative RBCs may be difficult to maintain in blood bank inventory.

**Study Design and Methods:**

We changed our blood bank laboratory policy to approve non‐alloimmunized Rh(D)‐negative patients to receive Rh(D)‐positive RBCs for routine transfusion under defined criteria. Those criteria included Rh(D)‐negative males (all ages) and females (aged >50 years) who were designated as do not resuscitate (DNR), either with or without intubation, in the electronic medical record.

**Results:**

From August 15, 2024 through August 15, 2025, a total of 204 Rh(D)‐negative patients met the above criteria and were approved to receive routine Rh(D)‐positive RBC transfusions. Within that group, 23 patients received Rh(D)‐positive RBCs. The remaining patients either did not require transfusion or were issued Rh(D)‐negative RBC units. Since implementing this practice, a total of 68 Rh(D)‐negative units were conserved during this time frame. Notably, 28 of the 68 units (41%) were type O, Rh(D)‐negative.

**Discussion:**

Rh(D)‐positive RBCs can be routinely given to non‐alloimmunized Rh(D)‐negative patients who are not at risk for developing hemolytic disease of the fetus and newborn (HDFN). By creating clear guidelines for the routine administration of Rh(D)‐positive RBCs to patients who are not at risk for HDFN, the inventory of Rh(D)‐negative RBC units can be directed to those patients who would most benefit from this limited resource.

**Abbreviations**
CPR: cardiopulmonary resuscitation
DNR: do not resuscitate
HDFN: hemolytic disease of the fetus and newborn
RBC: red blood cell

## 1 INTRODUCTION

In the last decade, the transfusion medicine community has recognized that the chronically short supply of Rh(D)‐negative red blood cell (RBC) units, particularly type O, is due, in part, to overutilization at the hospital level.^1^, ^2^ While approximately 6.9% of American blood donors are group O Rh(D)‐negative, the proportion of group O Rh(D)‐negative RBC units distributed by blood suppliers is far higher, reaching 12.1% in 2023.^3^ As such, strategies to reduce hospital reliance on Rh(D)‐negative RBC units are being emphasized by blood suppliers.

Most transfusion services have defined policies or procedures that support the emergent transfusion of Rh(D)‐positive RBC units to Rh(D) negative patients or patients of unknown ABO and Rh type.^4^ Transfusion services may also have defined policies or procedures for the transfusion of Rh(D)‐positive RBC units to Rh(D)‐negative patients during times of Rh(D)‐negative RBC inventory shortage. Less common, however, are policies or procedures that allow for the routine transfusion of Rh(D)‐positive RBC units to Rh(D)‐negative patients, even in times of adequate Rh(D)‐negative RBC inventory.

Routinely transfusing Rh(D)‐positive RBC units to Rh(D)‐negative patients primarily raises concerns about Rh(D) alloimmunization. The consequences of Rh(D) alloimmunization are most clinically significant in women of childbearing potential, that is, the concern for hemolytic disease of the fetus and newborn (HDFN) in future pregnancies. However, for patients of non‐childbearing potential, the consequences of Rh(D) alloimmunization are largely insignificant. That is, if a patient of non‐childbearing potential were to become.

Rh(D) alloimmunized, no future pregnancies are at risk and all future transfusion needs would simply be fulfilled with Rh(D)‐negative RBC units.

Given the above, our blood bank laboratory considered possible policies that would allow for the routine transfusion of Rh(D)‐positive RBC units to Rh(D)‐negative patients, even in times of adequate Rh(D)‐negative RBC inventory. This would allow the blood bank laboratory to reduce the number of Rh(D)‐negative RBC units used for Rh(D)‐negative patients of non‐childbearing potential. Our blood bank laboratory chose to focus our efforts on non‐alloimmunized Rh(D)‐negative patients of non‐childbearing potential who were designated as do not resuscitate (DNR) in the electronic medical record. Notably, DNR/DNI and DNR, may intubate are the code status options available in our hospital system in the electronic medical record (EMR).

DNR, do not attempt resuscitation (DNAR), and allow natural death (AND) are different names for “code status” orders that equivalently direct the health care team to withhold resuscitative measures in the event they are necessary, as decided by a patient and/or the patient's family.^5^ If a DNR/DNAR/AND order is placed in a patient's EMR, it can be assumed that conversations were completed with the patient and/or their family and that the relevant parties understand that the risks and burdens of cardiopulmonary resuscitation (CPR) outweigh the possible and unlikely benefits.^6^ While relevant conversations can be completed with a patient and/or their family at any time during life and during care, conversations about the utility of CPR are most common at the end of life. Indeed, a DNR/DNAR/AND order in the medical record most commonly designates the decision to emphasize end‐of‐life care. That said, DNR/DNAR/AND orders do not preclude blood transfusion support, and transfusion is not uncommonly part of the patient care plan at end‐of‐life.

Our blood bank laboratory reasoned that possible Rh(D) alloimmunization in Rh(D)‐negative patients of non‐childbearing potential who were designated as DNR would be of minimal clinical consequence. Although a theoretical risk of alloimmunization exists, many of these patients were unlikely to become alloimmunized to the Rh(D) antigen given their often‐diminished clinical status and/or immunosuppression. It was also possible that many of these patients would expire before alloimmunization could even occur. As such, as a means by which to conserve Rh(D)‐negative RBC inventory, we approved Rh(D)‐negative patients who fit inclusion criteria and were designated as DNR to routinely receive Rh(D)‐positive RBC units.

## 2 STUDY DESIGN/METHODS

Thomas Jefferson University Hospital is an academic medical center, and our transfusion service supports this 862 bed, Level 1 trauma center, utilizing approximately 20,000 RBC transfusions annually. The hospital is also a multi‐organ transplant center and manages nearly 3000 obstetric deliveries each year, serving a large and diverse patient population. Starting on August 15, 2024, we changed our blood bank laboratory policy to approve non‐alloimmunized Rh(D)‐negative patients to receive Rh(D)‐positive RBCs for routine transfusion under defined criteria. Those criteria included Rh(D)‐negative males of all ages and females aged >50 years, who were designated as DNR/DNI and DNR, may intubate in the EMR (Epic, Verona, Wisconsin). DNAR and AND are not code status options in our institution's EMR. These patients were prospectively identified by periodic reviews of current inpatient lists in the EMR by the blood bank laboratory Quality Coordinator. Real‐time changes in patient code status are not available for review with our current computer systems. Using a report generated in Qlik (King of Prussia, Pennsylvania), physician orders extracted from the electronic medical record were analyzed; patients designated as DNR/DNI and DNR, may intubate were identified. Once identified, patients had a “note to tech” placed in the laboratory information system (LIS; SoftBank, Clearwater, Florida) which then allowed the routine issuance of Rh(D)‐positive RBCs. This policy change was discussed during our quarterly transfusion committee meeting, receiving full support from attendees.

While this policy remains in place currently, data were reviewed from August 15, 2024 through August 15, 2025. During this year window, we reviewed the number of Rh(D)‐negative patients designated as DNR who were subsequently approved to receive routine Rh(D)‐positive RBC transfusions. The gender and ABO and Rh type of these patients were recorded. The number of Rh(D)‐positive RBC units transfused to these Rh(D)‐negative patients was quantified, thus representing the number of Rh(D)‐negative RBC units conserved by this new policy. No further clinical or laboratory patient information was collected.

## 3 RESULTS

From August 15, 2024 through August 15, 2025, a total of 204 Rh(D)‐negative patients were identified as meeting the above stated criteria and approved to receive routine Rh(D)‐positive RBC transfusions. Within that group, 23 patients received Rh(D)‐positive RBCs (Table 1). The remaining patients either did not require transfusion or they were issued Rh(D)‐negative RBC units. A total of 68 Rh(D)‐negative RBC units were conserved during the above time frame due to this practice. Notably, 28 of the 68 Rh(D)‐negative RBC units conserved (41%) were type O, Rh(D)‐negative. Thirty‐nine of the 68 Rh(D)‐negative RBC units conserved (57%) were type A, Rh(D)‐negative. One of the 68 Rh(D)‐negative RBC units conserved (2%) was type B, Rh(D)‐negative. There were no instances of a patient being misidentified as DNR by the blood bank laboratory Quality Coordinator and thus inappropriately receiving Rh(D)‐positive RBCs. There were no identified instances where a patient's code status was changed from DNR/DNI or DNR, may intubate, to full code.

**TABLE 1:** Rh(D)‐negative patients receiving Rh(D)‐positive and Rh(D)‐negative red blood cell transfusions.

Patient	Gender	ABO/Rh type	# Rh(D)‐positive RBC units transfused	# Rh(D)‐negative RBC units transfused
1	M	A neg	1 A pos	1 O neg
2	F	O neg	2 O pos	2 O neg
3	F	O neg	1 O pos	1 O neg
4	F	A neg	3 A pos	2 A neg
5	M	A neg	4 A pos	2 A neg
6	F	AB neg	9 A pos	1 A neg, 1 AB neg
7	F	A neg	1 A pos	None
8	F	A neg	1 A pos	1 A neg
9	F	A neg	3 A pos	None
10	F	A neg	1 A pos	None
11	M	O neg	3 O pos	1 O neg
12	F	O neg	3 O pos	None
13	F	A neg	1 A pos	None
14	M	A neg	2 A pos	None
15	M	O neg	4 O pos	3 O neg
16	F	A neg	1 A Pos	None
17	M	A neg	1 A pos	2 A neg
18	M	O neg	6 O pos	None
19	M	A neg	6 A pos	None
20	M	O neg	2 O pos	None
21	M	A neg	5 A pos	1 A neg
22	M	O neg	7 O pos	None
23	F	B neg	1 B pos	1 B neg

## 4 DISCUSSION

The transfusion of Rh(D)‐positive blood products to Rh(D)‐negative patients of non‐childbearing potential is not uncommon practice in transfusion medicine. For example, trauma patients of non‐childbearing potential are often transfused with type O, Rh(D)‐positive RBCs or whole blood prior to ABO/Rh type determination. Rh(D)‐positive platelet units are frequently, if not routinely, transfused to Rh(D)‐negative patients of non‐childbearing potential. Rh(D)‐positive RBC units may also be transfused to Rh(D)‐negative patients of non‐childbearing potential during times of Rh(D)‐negative RBC inventory shortage. The risk of Rh(D) alloimmunization due to the above practices is considered either inconsequential and/or reasonable, given inventory constraints.

By extension, our blood bank laboratory reasoned that routinely transfusing Rh(D)‐positive RBC units to Rh(D)‐negative patients of non‐childbearing potential who were designated as DNR in the EMR also would be of minimal clinical consequence. Patients designated as DNR often still require transfusion support, but they can generally be presumed less likely to become Rh(D) alloimmunized. While the theoretical risk of antibody formation remains, this population is less likely to undergo alloimmunization due to their relatively suppressed immune status and their likely shortened clinical trajectory, which may limit the opportunity to develop alloantibodies to the Rh(D) antigen. Further, as our blood bank laboratory only applied this policy to patients designated as DNR who were also of non‐childbearing potential, Rh(D) alloimmunization, if it occurred, would have no implications for future pregnancies.

While our blood bank laboratory chose to include DNR patients in this policy, we intentionally chose to exclude patients designated in the EMR as receiving “comfort measures.” Patients receiving “comfort measures” may be facing death more imminently than those designated as DNR. Patients receiving “comfort measures” may also less frequently receive transfusion support. As such, it was felt that the routine administration of Rh(D)‐positive RBCs to Rh(D)‐negative patients receiving “comfort measures” would not make a meaningful change in Rh(D)‐negative RBC utilization.

As implemented, over the course of 1 year, this blood bank laboratory policy conserved 28 type O, Rh(D)‐negative RBC units. For reference, our blood bank laboratory's optimal inventory for type O, Rh(D)‐negative RBC units is 30 units. Similarly, this blood bank laboratory policy conserved 39 type A, Rh(D)‐negative RBC units. Our blood bank laboratory's optimal inventory for type A, Rh(D)‐negative RBC units is 20 units. Thus, we believe that this blood bank laboratory policy enabled the meaningful conservation of Rh(D)‐negative RBC unit inventory. We also postulate that if other transfusion services implemented this policy, or a policy of similar spirit, the chronically short supply of Rh(D)‐negative RBC units would likely abate. Of note, the College of American Pathologists recently implemented a new checklist item TRM.40707, which requires the blood bank laboratory to have processes to conserve group O Rh(D)‐negative RBCs.^7^ Implementing a policy such as we describe may help other blood bank laboratories fulfill this stewardship requirement. Addressing the limited inventory of Rh(D)‐negative RBC units requires that the transfusion medicine community make incremental, yet meaningful, changes to their transfusion practices.

Table 1 emphasizes that Rh(D)‐negative patients who meet the specified criteria did not receive Rh(D)‐positive RBC units for all ordered RBC transfusions. That is, while our blood bank laboratory policy *allows* Rh(D)‐negative patients who meet the specified criteria to receive Rh(D)‐positive RBC units, this is not *required*. Rh(D)‐negative patients who meet the specified criteria sometimes received RBC transfusions at affiliate hospitals that were not participating in this initiative, and these patients were therefore transfused Rh(D)‐negative RBC units. If our blood bank laboratory had short‐dated Rh(D)‐negative RBC units, these would be transfused to Rh(D)‐negative patients meeting the specified criteria in lieu of longer‐dated Rh(D)‐positive RBC units.

Additionally, Rh(D)‐negative RBC units were also sometimes transfused to Rh(D)‐negative patients meeting the specified criteria because of push back from transfusionists. Some transfusionists have questioned or even refused to transfuse Rh(D)‐positive RBC units to Rh(D)‐negative patients meeting the specified criteria because of concerns about incompatibility, possible transfusion reactions, or unauthorized “research.” In these instances, the covering blood bank physicians have attempted to re‐educate the transfusionists on why transfusing Rh(D)‐positive RBC units to Rh(D)‐negative patients meeting the specified criteria is reasonable and safe. Although bedside transfusionists occasionally raised concerns with the blood bank physicians, the clinicians responsible for ordering these transfusions consistently demonstrated a clear understanding of the rationale for administering Rh(D)‐positive RBC units to Rh(D)‐negative patients and expressed support for this approach.

In response to the above transfusionist concerns, our blood bank laboratory developed a document in an SBAR (Situation, Background, Assessment, Recommendation) format to be distributed in paper format to transfusionists whenever Rh(D)‐positive RBCs are issued to Rh(D)‐negative patients meeting the specified criteria. This document, included as an electronic supplement to this publication, reviews why transfusing Rh(D)‐positive RBCs to Rh(D)‐negative patients is both reasonable for inventory management and safe for patient care. Our blood bank physicians have offered to provide didactic education to transfusionists through hospital nursing educators; however, this has not yet resulted in a scheduled educational session.

A possible limitation of our implemented blood bank laboratory policy is that we chose not to perform, nor collect data on, subsequent patient antibody testing to determine if Rh(D) alloimmunization occurred following a transfusion of Rh(D)‐positive RBCs. Indeed, we made the very intentional decision not to pursue this data, not only to preclude the need for institutional review board (IRB) oversight, but also to emphasize that Rh(D) alloimmunization in Rh(D)‐negative patients of non‐childbearing potential is of minimal clinical consequence. This is particularly true in a patient population designated as DNR in the medical record. Again, the purpose of our implemented blood bank laboratory policy was to conserve Rh(D)‐negative RBC units, not to explore the well‐established, but clinically insignificant, risk of Rh(D) alloimmunization in Rh(D)‐negative patients of non‐childbearing potential.

Our implemented blood bank laboratory policy underscores that Rh(D)‐negative RBC units can be conserved by routinely transfusing Rh(D)‐positive RBCs to non‐alloimmunized Rh(D)‐negative patients who are not at risk for developing HDFN. By creating clear criteria or guidelines for the routine administration of Rh(D)‐positive RBCs to Rh(D)‐negative patients who are not at risk for HDFN, transfusion medicine services can optimize the use of Rh(D)‐negative blood units.

## CONFLICT OF INTEREST STATEMENT

The authors declare no conflicts of interest.

## Supporting information


**Data S1.** Supporting Information.

## Data Availability

Research data are not shared.
