# Supplementary material for: Routine transfusion of Rh(D)‐positive RBCs to Rh(D)‐negative patients designated as do not resuscitate conserves Rh(D)‐negative red blood cell inventory
Source: Transfusion. 2026 Apr 12;66(6):1108–12. doi: 10.1111/trf.70209 (PMC13250371; doi:10.1111/trf.70209)
Supplement: Supplementary file 1 — Data S1. Supporting Information. [file TRF-66-1108-s001.docx]

**Situation and Background:** A great minority of the US population/blood donors are Rh (D) negative. As such, Rh (D) negative red blood cell (RBC) products are often challenging to keep in blood bank inventory. Rh (D) negative RBCs should always be provided to Rh (D) negative women of childbearing potential (defined as age ≤ 50 years) to prevent them from making anti-D and prevent the possibility of anti-D mediated hemolytic disease of the fetus and newborn (HDFN). Other Rh (D) negative patient populations (ie. men or women of non-childbearing potential) may receive Rh (D) positive RBCs without concern for HDFN.

**Assessment:** Rh (D) negative RBC inventory continues to be challenge for the blood bank. Steps are needed to further preserve Rh (D) negative RBC inventory for Rh (D) negative women of childbearing potential.

**Recommendation:**

1. As blood bank inventory dictates, Rh (D) positive RBCs will be transfused to some Rh (D) negative patients (ie. men or women of non-childbearing potential).
2. Rh (D) negative patients who are men or women of non-childbearing potential with a code status of Do Not Resuscitate and/or Do Not Intubate (DNR and/or DNI) will be transfused Rh (D) positive RBCs.
3. Rh (D) positive RBCs may safely be transfused to Rh (D) negative patients who have not made anti-D. These RBC units are crossmatch compatible, as indicated on the unit tag.
4. If you have further questions, please call the blood bank (56356) and ask to be directed to a blood bank physician.
